# Diverse RNAs in adipose-derived extracellular vesicles and their therapeutic potential

**DOI:** 10.1016/j.omtn.2021.08.028

**Published:** 2021-08-28

**Authors:** Pengyu Hong, Mei Yu, Weidong Tian

**Affiliations:** 1 State Key Laboratory of Oral Disease & National Clinical Research Center for Oral Diseases West China School of Stomatology, Sichuan University, Chengdu, China; 2 National Engineering Laboratory for Oral Regenerative Medicine, West China School of Stomatology, Sichuan University, Chengdu, China; 3 Engineering Research Center of Oral Translational Medicine, Ministry of Education, Sichuan University, Chengdu, China; 4 Department of Oral and Maxillofacial Surgery, West China Hospital of Stomatology, Sichuan University, Chengdu, China

**Keywords:** RNAs, adipose tissue, extracellular vesicles, exosomes, therapeutic potential

## Abstract

Adipose tissue, which is considered an energy storage and active endocrine organ, produces and secretes a large amount of adipokines to regulate distant targets through blood circulation, especially extracellular vesicles (EVs). As cell-derived, membranous nanoparticles, EVs have recently garnered great attention as novel mediators in establishing intercellular communications as well as in accelerating interorgan crosstalk. Studies have revealed that the RNAs, including coding RNAs (messenger RNAs) and noncoding RNAs (long noncoding RNAs, microRNAs, and circular RNAs) are key bioactive cargoes of EV functions in various pathophysiological processes, such as cell differentiation, metabolic homeostasis, immune signal transduction, and cancer. Moreover, certain EV-contained RNAs have gradually been recognized as novel biomarkers, prognostic indicators, or even therapeutic nanodrugs of diseases. Therefore, in this review, we comprehensively summarize different classes of RNAs presented in adipose-derived EVs and discuss their therapeutic potential according to the latest research progress to provide valuable knowledge in this area.

## Graphical abstract

**Figure:**
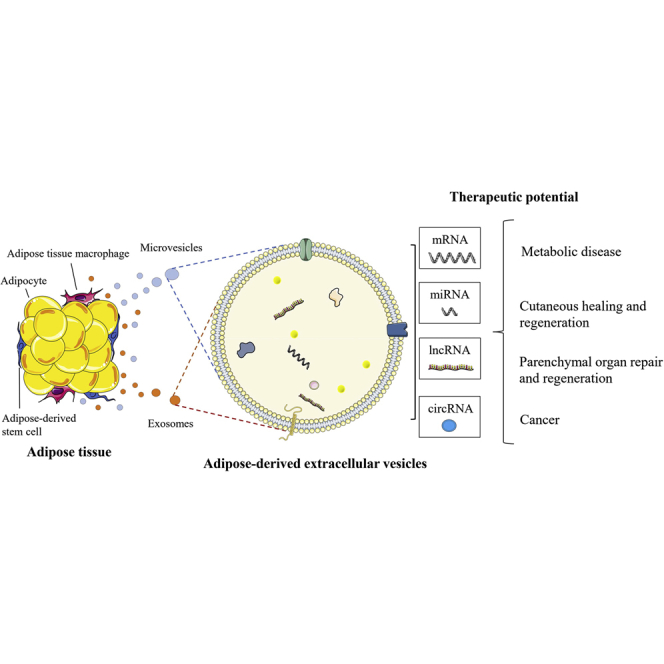

## Teaser

In this review, Hong et al. focuses in detail on introducing the diverse RNAs (mRNAs, miRNAs, lncRNAs, and circRNAs) of adipose-derived extracellular vesicles and on summarizing their effects and mechanisms in different disease models, as well as their therapeutic potential.

## Introduction

Adipose tissue is generally composed of many heterogeneous cell types, such as adipose-derived stem cells (ASCs), preadipocytes, mature adipocytes, as well as fibroblasts, nerve cells, macrophages, endothelial cells, and muscle cells.^1^ Studies have demonstrated that adipose tissue should be regarded not only as an energy-storage organ but also as an endocrine organ that synthesizes and releases factors, especially “adipokines,” which function as secretory factors to communicate with other metabolic organs and regulate whole-body energy homeostasis.^2^^,^^3^ Certain soluble adipokines, such as leptin and adiponectin secreted by adipose-derived cells have been well studied.^4^ In addition to those traditional, soluble mediators, recent studies have shown that the RNAs transported by extracellular vesicles (EVs) can also exert adipokine-like functions.^5^ For example, micro RNAs (miRNAs) of EVs derived from adipose tissue can act as endocrine messengers that facilitate communication that ranges from nearby cells to distant tissues, and they are, therefore, considered a novel kind of adipokine.^2^^,^^6^

Importantly, the exploration and recognition of RNAs in EVs has progressed immensely because of the comprehensive use of high-throughput RNA sequencing and validation of quantitative polymerase chain reaction (qPCR), even though they are present in small amounts.^7^ Hence, studies have gradually acknowledged and appreciated more-functional RNAs mediated by EVs, which represent a novel mode of intercellular communication and may act as novel factors to exert potential therapeutic benefits.^8^ However, the RNA pools carried by EVs come from adipose-derived tissues and cells that have not yet been completely characterized. Therefore, in this review, we will illustrate the diverse RNAs in adipose-derived EVs and highlight their therapeutic potential according to the most-recent relevant literature to summarize practical and valuable knowledge in this area.

## Characteristic and biogenesis of extracellular vesicles

EVs are defined as nanoparticles released from cells and are delimited by a lipid bilayer, which does not have a functional nucleus and cannot replicate.^9^ EVs can be secreted by almost all cells and have been extracted from several body tissues, as well as varieties of body fluids, including urine, saliva, plasma, semen, breast milk, and lymph fluids.^10^^,^^11^ According to the size and biogenesis of EVs, there are three major types that produce them: exosomes, microvesicles (MVs), and apoptotic bodies.^7^ Here, in this review, we mainly discuss the first two types of EVs. Exosomes are produced from the endosomal pathway and are secreted as particles ranging from 40 nm to 160 nm in diameter.^12^ By contrast, MVs are vesicles produced by direct outward budding of the cell membrane and are on the order of 50–1,000 nm in diameter.^13^

Exosomes and MVs have different modes of biogenesis. Exosomes are generally formed by invagination of the endosomal membrane forming intraluminal vesicles (ILVs) within multivesicular bodies (MVBs). First, early endosomes, formed by lipid raft domains of the plasma membrane, mature into late endosomes with the assistance of the Golgi complex. Then, the bilayer membrane of late endosomes produces ILVs, which accumulate in their lumen. Lastly, the ILVs fuse with the cell membrane and are released into the extracellular environment and are known as exosomes.^11^^,^^14^ Regarding the formation of MVs, they are related to the molecular redistribution of the cell membrane bilayer, which includes phospholipids and proteins as well as Ca^2+^ levels.^15^ These processes generally include two major pathways: those dependent on the endosomal sorting complex required for transport (ESCRT), or those that are independent of the ESCRT.^15^ The ESCRT machinery is said to act in a stepwise manner, requiring a sequential performance of the four ESCRT subunits (ESCRT-0, -I, -II, and -III), associated proteins (ALG2-interacting protein X [ALIX] and vacuolar protein sorting-associated protein 4 [VPS4]), and lipid rafts.^16^ ESCRT0 and ESCRTI complexes cluster transmembrane cargo into the vesicles and recruit them via ESCRTII; then, the ESCRTIII subunits drive fission and budding of the membranes, which finally produces exosomes.^15^ In addition, the ESCRT is also involved in MV formation and the last phase of their release. Studies indicate that arrestin domain-containing protein 1 (ARRDC1), an adaptor protein, recruits ESCRT proteins of the tumor susceptibility gene 101 (TSG101) and VPS4 to the cell membrane, which prompt them to bud out from the cell membrane and share many of the same functional cargoes as that of exosomes.^17^

Research has shown that EVs extensively participates in physiological and pathological conditions.^18^, ^19^, ^20^ The main components of EVs contain a wide range of cargoes (for example: proteins, RNAs, and lipids), which can be transferred between cells as a means of cell-cell communication at both paracrine levels and systemic levels.^21^^,^^22^ Of note, some RNA cargoes are found to be selectively incorporated into EVs.^14^ The transcriptomes of different cell types, even different cell differential states, are all partially reflected in their extracellular vesicle RNA cargo.^23^^,^^24^ For example, a recent study indicated that miRNAs contributing to brown fat cell differentiation are enriched in EVs derived from ASCs during beige adipogenic differentiation compared with EVs derived from ASCs during proliferation.^24^ When EVs are internalized, the complex, bioactive cargoes from their progenitor cells elicit diverse and pleiotropic responses in recipient cells, including angiogenesis, adipogenesis, osteogenesis, immune modulation, and cancer progression.^25^, ^26^, ^27^, ^28^ Because of the multiple functions of EVs, these nanovesicles, whether they are derived from tissue or cells, are collectively reported to have therapeutic effects on tissue repair, wound healing, immune modulation, diabetes, oncotherapy, and other diseases.^29^, ^30^, ^31^, ^32^, ^33^ Therefore, EVs have already displayed their critical and crucial role in cell-free therapeutic strategies, with huge potential, and have also aroused great concern in biomedical frontiers.

## Diverse RNAs in adipose-derived extracellular vesicles

**Figure 1:**
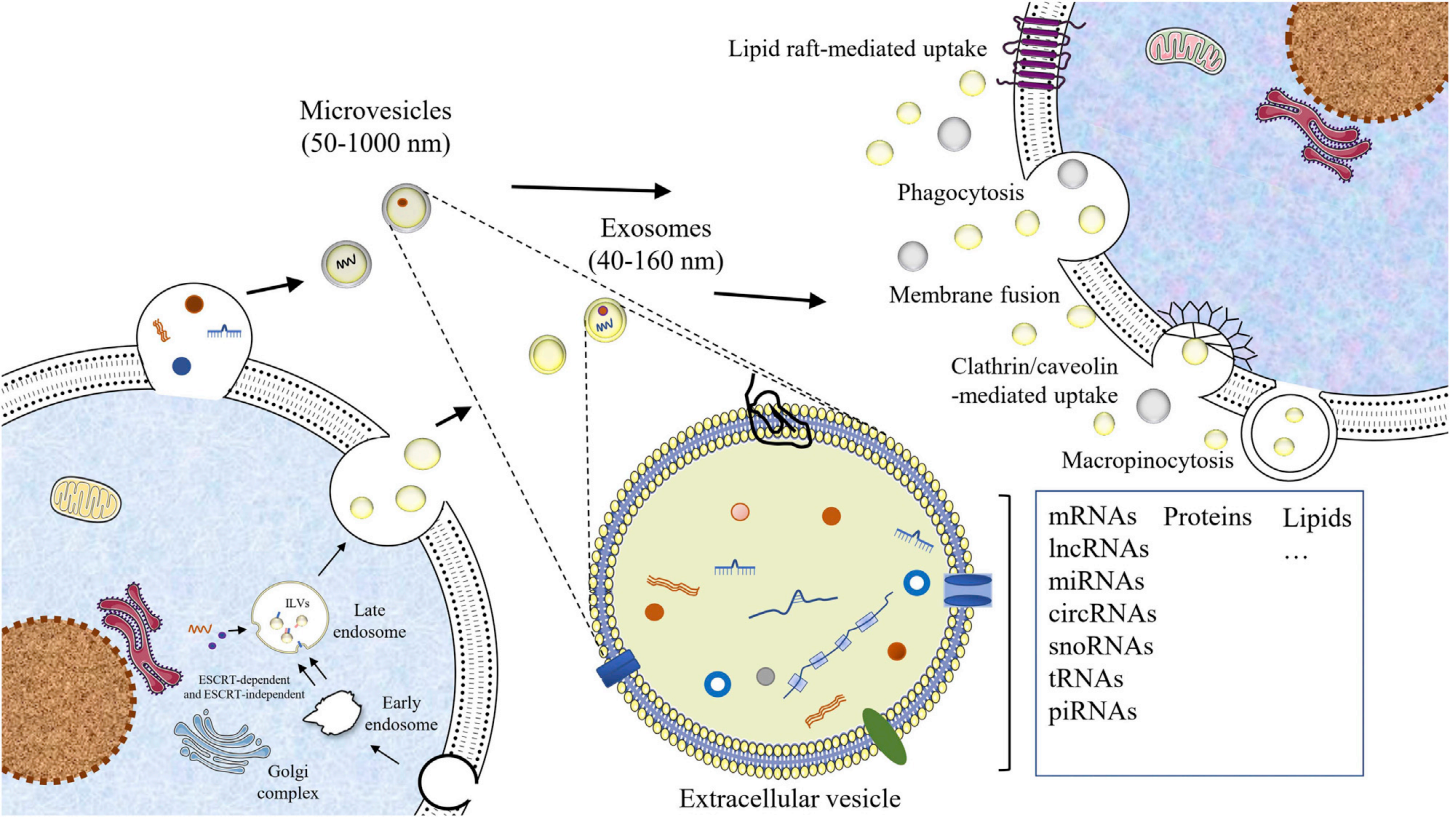
The contents and uptake of extracellular vesicles Exosomes (40–160 nm) are formed by multi-vesicular bodies (MVBs) fusing with the cell membranes and releasing extracellularly. Microvesicles (50–1,000 nm) are formed by outward budding of the plasma membrane. EVs have been shown to be internalized by cells through clathrin/caveolin-mediated endocytosis, phagocytosis, macropinocytosis, lipid raft-mediated uptake, or direct membrane fusion; upon which, the EV cargos (RNAs, proteins, lipids, etc.) are released into the recipient cells.

In recent years, the exploration of diverse RNAs in adipose-derived EVs has progressed extensively because they are being investigated as novel biomarkers, pathophysiological mediators, and potential therapeutic drugs.^2^^,^^34^^,^^35^ These RNA populations include messenger RNAs (mRNAs), miRNAs, long noncoding RNAs (lncRNAs), circular RNAs (circRNAs), transfer RNAs (tRNAs), small nucleolar RNAs (snoRNAs), and PIWI-interacting RNAs (piRNAs).^12^^,^^35^, ^36^, ^37^, ^38^ Relevant to the packaging of RNA cargoes into EVs, emerging studies have demonstrated that certain RNAs in EVs are selectively enriched compared with their parental cells.^2^^,^^39^ There is evidence that this kind of RNA sorting is a rigid and regulated process.^40^ For instance, specific RNA sequences are retained and then packaged by EVs, which are generally transferred by RNA-binding proteins (RBPs), including the heterogenous nuclear ribonucleoprotein (hnRNP) family, Argonaute 2 (Ago2), Y-box-binding protein 1 (YB-1), NOP2/Sun domain family member 2 protein (NSUN2), and others.^41^^,^^42^ Furthermore, multiple routes of EV uptake have been reported. RNAs containing EVs can enter recipient cells via clathrin/caveolin-mediated endocytosis, macropinocytosis, phagocytosis, lipid raft-mediated uptake, or direct membrane fusion (Figure 1).^14^^,^^19^^,^^43^ Although certain RNA functions in adipose-derived EVs are not yet known, the fact that these RNAs are able to be diverted from donor cells to recipient cells has suggested an important and indispensable communication system between cells. Therefore, in the following section, this review will discuss mRNAs, miRNAs, lncRNAs, and circRNAs contained in adipose-derived EVs.

## mRNA

Early pioneer work has established that mRNAs are first synthesized in the cell nucleus, where they undergo splicing and modification, and then, they are transported to the cytoplasm, where they serve as protein translational templates before being degraded. Interestingly, Valadi et al.^44^ first found that there were mRNAs and miRNAs contained in exosomes, which could be delivered to other cells, and had different functional roles in those new locations. Recent studies have shown that full-length or fragmented mRNAs in various EVs can be carried as genetic cargoes and participate in a variety of physiological and pathological processes.^7^^,^^45^ For example, previous RNA sequencing and bioinformatic analyses have revealed that EVs that come from adipose-derived stem cells (EV-ASCs) preferentially express mRNA encoding for transcription factors that are involved in angiogenesis and adipogenesis (e.g., hairy and enhancer of split-1 [HES1], CCAAT/enhancer binding protein [CEBP]-alpha [CEBPα], transcription factor-4 [TCF4], and Kruppel-like factor-7 [KLF7]), Golgi apparatus proteins, and proteins involved in transforming growth factor β (TGF-β) signaling.^36^^,^^46^ RNA sequencing also found that 255 mRNAs, more than 70% of which were related to transcription factors, were selectively upregulated in EV-ASCs versus ASCs.^47^ These data provide insights into the principal capabilities of EV-ASCs with respect to their potential to modulate molecular networks and interactions in the recipient cells.

Moreover, Müller et al.^48^ found that adipocyte-derived EVs contained specific transcripts and miRNAs, which could both be transferred into recipient adipocytes and were involved in the upregulation of lipogenesis and cell size. In addition, adipocyte-derived EVs were also able to transfer adipogenic mRNAs (peroxisome proliferators-activated receptors-gamma [PPARγ], leptin, CEBPα. and CEBPδ) and anti-osteoblastic miRNAs to osteoblasts, which results in the decreased expression of osteocalcin and osteopontin.^49^ Furthermore, mRNAs can also be engineered and modified in target adipose-derived EVs as novel genetic drugs and vehicles. For example, Chen et al.^50^ transfected a glial cell line-derived neurotrophic factor (GDNF; a neurotrophic factor that protects nigral dopaminergic neurons) into ASCs and successfully isolated GDNF-ASC-EVs, and then, identified the ability of those EVs to exert cytoprotective effects on human umbilical vein endothelial cells (HUVECs) by reducing hypoxia/serum deprivation (H/SD) injury. The migration, angiogenesis, and apoptosis-resistance capacities of HUVECs were all increased. Moreover, GDNF-ASC-EVs promoted SIRT1 signaling mechanistically, accompanied by increased levels of phosphorylated endothelial nitric oxide synthase.^50^ Collectively, mRNAs encapsulated in adipose-derived EVs not only can be directly transferred but also can be artificially modified before transfer; both of which may contribute to biological, functional changes in recipient cells.

## miRNA

miRNAs are a class of small noncoding RNAs (∼22 nt), highly conserved, and single-stranded RNAs that can modulate the translational efficiency of their target mRNAs in various cell types.^51^ Studies have shown that miRNAs generally contain 6- to 8-nt seed sequences that can integrate with the 3′ untranslated regions (UTRs) of mRNAs.^52^^,^^53^ Binding of these miRNAs to target mRNAs recruits those mRNAs to the RNA-induced silencing complex (RISC) and, then, triggers mRNA silencing and degradation.^54^ Moreover, miRNAs are not only formed and exert their functions inside of cells but also can be released into the circulation and body fluids to participate in various intercellular signaling.^2^ Of note, miRNAs are found to be packaged within EVs and released by many types of cells and tissues, especially from adipose sources; of which, miRNAs serve as a new class of endocrine factors and adipokines.

One study conducted miRNA microarray analysis by comparing ASC-derived MVs and ASCs. Some miRNAs were found to be produced and selectively packaged into EVs; of which, nine miRNAs were downregulated in ASC-derived MVs, whereas 23 miRNAs were upregulated compared with ASCs.^55^ For example, two miRNA members of the let-7 family (let-7i-5p and let-7f-5p), which have been proven to promote angiogenesis, were more highly expressed in ASCs than they were in ASC-derived MVs.^55^^,^^56^ In addition, different miRNA profiles have been reported to be of different origins in adipose-derived EVs, implicating their distinct and indispensable roles in regulating biological processes. Zhang et al.^35^ profiled the miRNAs in exosome-like vesicles derived from adipose tissue (Exo-AT) and exosome-like vesicles derived from ASCs via using high-throughput sequencing. The results revealed 45 miRNAs were highly expressed in Exo-AT compared with the exosome-like vesicles derived from ASCs; of which, 14 miRNAs (e.g., miR-30a-5p and miR-148a-3p) were associated with adipogenesis, eight miRNAs (e.g., miR-93-5p and miR-150-3p) were demonstrated to repress osteoblastic differentiation of mesenchymal stem cells (MSCs), nine miRNAs (e.g., miR-150-3p and miR-126a-3p) were related to angiogenesis, and four miRNAs (such as miR-224) were associated with metabolic regulation.^35^ In addition, recent studies have demonstrated that adipose tissue macrophages (ATMs) are at the root of the aberrant inflammation in hypertrophic, chronic adipose-tissue inflammation, and they may exert critical functions in type 2 diabetes and insulin resistance.^57^^,^^58^ Moreover, evidence has revealed that exosomes derived from ATMs (Exo-ATMs) transferred miRNAs to insulin target cells, and deep sequencing of miRNAs from lean and obese Exo-ATMs have identified more than 500 miRNAs; among which, a set of miRNAs (such as miR-155), which was related to insulin sensitivity and glucose tolerance, was differentially expressed in obese versus lean Exo-ATMs.^6^

Because miRNAs have displayed great potential for therapeutic applications in altering gene expression locally and distantly, studies to date have employed a variety of methods to engineer and modify certain amounts of miRNAs transferred by EVs to achieve their aims. For example, Qu et al.^59^ demonstrated that exosomes derived from miR-181-5p-modifed ASCs could reduce liver fibrosis through activating autophagy. Exosomes derived from miR-199a- and miR-122-modified ASCs could effectively improve hepatocellular carcinoma (HCC) chemosensitivity by directly enhancing the sensitivity of HCC cells.^60^^,^^61^ In addition, exosomes from miR-146a-overexpressing ASCs have been proven to exert therapeutic effects in relieving acute myocardial infarction (AMI)-induced myocardial damage.^62^ Collectively, because engineered miRNAs are currently just in their beginning stages, use of adipose-derived EVs as natural miRNA delivery vehicles has aroused great interest and concern. With the extensive studies conducted in miRNAs of adipose-derived EVs, this novel domain is anticipated to become prosperous, along with growing knowledge of the production, enrichment, transfer, and specific functions of miRNAs delivered by adipose-derived EVs.

## lncRNA

lncRNAs are currently defined as RNAs greater than 200 base pairs (bp) without protein-coding capacity.^63^ This is a broad definition that comprises a heterogeneous class of RNA transcripts, including promoters, enhancers, snoRNAs, intergenic transcripts, and transcripts that overlap other transcripts in either sense or antisense orientation.^64^ lncRNAs are demonstrated to engage in a variety of biological processes, such as cell differentiation, tumorigenesis, angiogenesis, immune reactions, and epigenetic regulation.^65^, ^66^, ^67^, ^68^, ^69^ Moreover, delivery of certain lncRNAs by EVs can exert curative effects on many diseases, among all organ systems.^18^ Certain star lncRNAs from EVs (such as MALAT1 and H19) have been proven to be related to cell proliferation, apoptosis, adipogenesis, and tumorigenesis.^70^, ^71^, ^72^ Along with numerous types of lncRNAs found in EVs, there particular interest has been shown on the therapeutic potential for harnessing the internal mechanisms of this novel, intercellular type of RNA transfer.^18^

Currently, most lncRNA-related studies of adipose-derived EVs have concentrated on the interaction between miRNAs and mRNAs. Patel et al.,^73^ using RNA sequencing, demonstrated that the lncRNA MALAT1 in exosomes derived from ASCs (Exo-ASCs) affected not only mRNA expression but also the expression of noncoding RNAs, especially snoRNAs in a model of traumatic brain injury. On the other hand, the lncRNA MALAT1 in Exo-ASCs was demonstrated to promote fibroblast migration and angiogenesis in a wound-healing model.^74^ In addition, the adipocyte-derived exosomal lncRNA SNHG9 can directly bind to the tumor necrosis factor receptor 1 (TNFR1)-associated death domain protein (TRADD) mRNAs, which, thus, constituted an RNA dimeric inducible-silencing complex and reduced endothelial dysfunction of obese patients.^75^ Further analysis has suggested that lncRNAs transferred by EVs could serve as sponges for miRNAs, thus, changing the expression of mRNAs indirectly. Shao et al.^76^ first uncovered and compared the profiles of exosomes derived from ASCs under hypoxia (Hyp/Exo-ASCs) and Exo-ASCs via high-throughput sequencing. The results revealed that expression of lncGm37494 was much greater in Hyp/Exo-ASCs than it was in Exo-ASCs, and its overexpression enhanced the M1/M2 polarization of microglial by suppressing miR-130b-3p and, thus, increasing the expression of PPARγ.^76^

Although much has been learned concerning the critical roles of lncRNAs in different types of cells and tissues, as illustrated, studies concentrating on adipose-derived EVs are still, so far, limited. Further research is urgently needed to expand our understanding of these lncRNAs.

## circRNA

circRNAs are classified as a novel group of tissue-specific endogenous noncoding RNAs expressed by the eukaryotic transcriptome.^77^ They are characterized by covalently bonded chains, continuous circle structures without a 5′ cap or a 3′ poly-A tail and that are much more resistant to digestion by RNase R exonuclease than linear RNAs are.^78^, ^79^, ^80^ To date, research using high-throughput RNA sequencing and microarray analyses have recognized various circRNAs that are selectively packaged into EVs.^81^, ^82^, ^83^ Furthermore, because circRNAs were demonstrated to be both contained in, and enriched in, EVs, researchers have started to explore the extracellular functions of those circRNAs, which have been found to exert critical roles in tumor immunity, cardiovascular diseases, and diabetes.^84^^,^^85^

Similar to lncRNAs, most existing literature shows that circRNAs in adipose-derived EVs can interact with certain miRNAs and regulate the stability and translation of mRNA.^86^ Zhang et al.^38^ found that exosomes secreted from adipocytes can mediate the delivery of circ-deubiquitination (circ-DB), then suppress miR-34a, and activate the USP7/cyclin A2 signaling pathway. Shi et al.^87^ verified that overexpression of mmu_circ_0000250 increased the therapeutic effect of Exo-ASCs in accelerating wound healing in a model of diabetes by inhibiting expression of miR-128-3p and upregulating expression of SIRT1. Another study used bioinformatics analysis and a dual-luciferase reporter assay to predict and validate circHivep2 as being able to absorb miR-181a-5p to promote SOCS2 expression, and they subsequently used Exo-ASCs as a vehicle to deliver circHivep2 into animal models of epilepsy to enhance the therapeutic effects.^83^ In addition to this sponge-like function, circRNAs can combine with certain proteins to form circRNA-protein complexes (circRNPs) and perform regulatory effects.^88^ For example, circ_0075932, contained in adipocyte-derived exosomes, can directly bind with the RNA-binding protein Pumilio2 (PUM2), and upregulate Aurora A kinase, thereby motivating the nuclear factor κB (NF-κB) pathway.^89^ In general, circRNAs are more stable than linear RNAs are because they do not contain free ends and may not be disintegrated by exonucleases.^7^ Importantly, the widespread use of adipose-derived EVs has allowed more possibilities for the exploration of the circRNA functions on different cellular processes and cell fates.

## Therapeutic potential of RNAs in adipose-derived EVs

**Figure 2:**
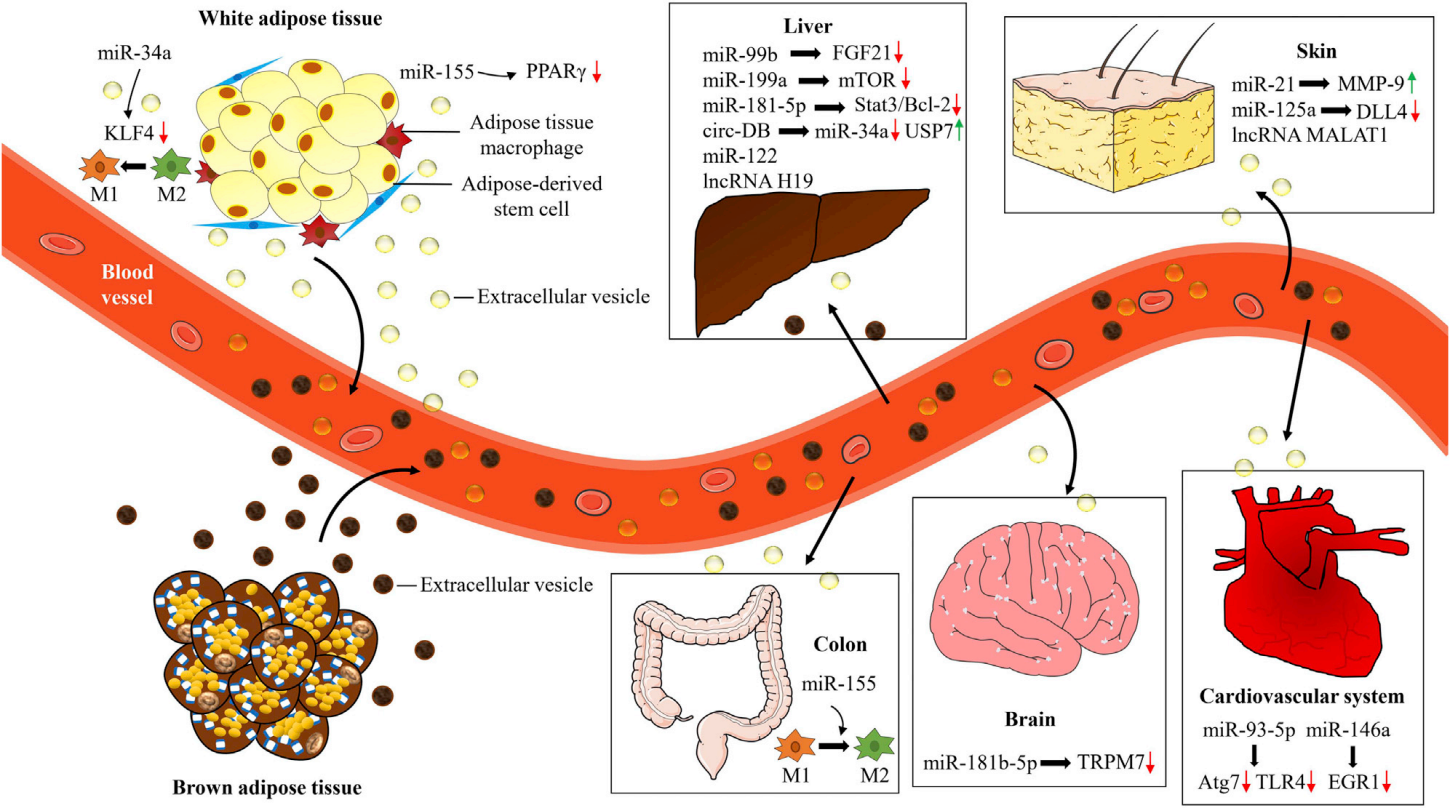
The RNAs of adipose-derived EVs enhance different organ crosstalk through blood circulation Recent studies have suggested that the RNAs of adipose-derived EVs can act as endocrine factors and exert their therapeutic potential to adjacent or distant organs, including the liver, skin, colon, brain, and cardiovascular system. Different functional RNAs are known to be secreted from adipose-derived EVs, and their related downstream genes in organ crosstalk are shown in this figure (lncRNA, long noncoding RNA; MALAT1, metastasis-associated lung adenocarcinoma transcript 1; Klf4, Krüppel-like factor 4; MMP-9, matrix metalloproteinase-9; STAT3, signal transducer and activator of transcription 3; Bcl-2, B cell lymphoma-2; FGF21, fibroblast growth factor 21; DLL4, delta-like ligand 4; circ-DB, circular RNA related to deubiquitylation (has_circ_0025129); EGR1, early growth response factor 1; TRPM7, transient receptor potential melastatin-subfamily member 7; USP7, ubiquitin-specific protease 7; TLR4, Toll-like receptor 4; mTOR, mechanistic target of rapamycin).

The therapeutic potential of RNAs in adipose-derived EVs has been proven in a variety of diseases, which is essential for progress in future clinical applications, such as metabolic dysfunction, tissue regeneration, tumor therapy, and other diseases (Figure 2). Adipose-derived EVs are distinct from traditional adipokines and can function in specific tissues because of their contents.^5^ Zhou et al.^90^ found that after intravenous injection, EVs derived from brown adipose tissue (EV-BAT) preferentially accumulated in the liver, which suggested that the liver could be one of its key target organs. In addition, a small proportion of EV-BATs circulated into the spleen, lung, white adipose tissue (WAT), and heart.^90^ Another study further concluded that adipose tissue was a major source of circulating exosomal miRNAs by comparing exosomes from the serum of mice with an adipose-tissue-specific knockout of the miRNA-processing enzyme Dicer (ADicerKO) and wild-type mice.^91^ Moreover, they found exosomes derived from BAT targeted liver metabolism more specifically and efficiently than exosomes derived from WAT, which suggested that this exosome-mediated organ crosstalk might be tissue specific.^90^^,^^91^

Studies, to date, have not only obtained and applied natural adipose-derived EVs to disease models but also attempted to develop engineered EVs to deliver therapeutic, modified biomolecules, especially by modifying the contents of certain RNAs.^92^ A few loading strategies have been developed and adopted so far, including genetic engineering of target RNAs in parent cells, thus altering the certain contents in the EVs and direct modification of EV properties after isolation, such as loading target RNAs by electroporation.^34^^,^^93^ These RNA-related biomimetic approaches elegantly use existing RNA-mimic synthesis techniques, natural cell-binding ability, and close protection against RNases degradation of EVs.^44^^,^^94^ However, regardless of whether EVs have been modified, wide organ-crosstalk functions and the easy availability of adipose tissue have led to numerous studies exploring RNAs of adipose-derived EVs in the field of disease treatment. Importantly, under various sources (e.g., EVs derived from adipose tissue under high-fat feeding) and circumstances, RNAs derived from adipose tissue are not always beneficial against diseases but, sometimes, make them worse.^6^ Only EVs containing RNAs with positive effects can serve as therapeutic candidates. Next, recent studies concerning the therapeutic potential of functional RNAs transferred by adipose-derived EVs will be specifically described in different clinical fields, which are summarized in Table 1, and abbreviations are summarized in Table 2.

**Table 1:** Functional delivery of RNAs by adipose-derived EVs

Process	Source	RNA	Function	Mechanism	Reference
Metabolic regulation	BAT	miR-99b	Maintaining metabolic homeostasis	Decreasing FGF21 mRNA levels and repressing activity of a FGF21 3′-UTR reporter	^91^
	ATMs	miR-155	Inhibiting insulin signaling and glucose tolerance	Directly suppressing its target gene PPARγ	^6^
	Mature adipocytes	miR-34a	Exacerbating obesity-induced, systemic inflammation, and metabolic dysregulation.	Suppressing M2 polarization by repressing the expression of Klf4	^101^
Cutaneous healing	ASCs	lncRNA MALAT1	Stimulating the healing of ischemic wounds	Promoting HDF cell migration and angiogenesis	^74^
	ASCs	miR-21	Accelerating wound healing	Enhancing the migration and proliferation of the HaCaT cells by increasing MMP-9 expression through the PI3K/AKT pathway	^109^
	ASCs	miR-125a	Promoting angiogenesis	Repressing expression of the angiogenic inhibitor DLL4	^113^
Parenchymal organ repair	ASCs	miR-93-5p	Reducing AMI-induced myocardial damage	Suppressing hypoxia-induced autophagy and inflammatory cytokine expression by targeting Atg7 and TLR4	^117^
	ASCs	miR-146a	Attenuating AMI-induced myocardial damage	Interacting with the 3′ UTR of EGR1 and suppressing posttranscriptional EGR1 expression	^62^
	ASCs	lncRNA H19	Attenuating acute liver failure	Promoting hepatocyte proliferation	^37^
	ASCs	miR-181-5p	Attenuating liver fibrosis	Downregulating Stat3 and Bcl-2 and activating autophagy in hepatic stellate cells; downregulating collagen I, vimentin, a-SMA, and fibronectin in CCl4-induced liver fibrosis	^59^
	Visceral adipose tissue	miR-155	Promoting intestinal inflammation	Promoting macrophage M1 polarization	^119^
	ASCs	miR-181b-5p	Promoting cerebral vascular remodeling	Promoting angiogenesis of brain microvascular endothelial cells after oxygen-glucose deprivation via miR-181b-5p/TRPM7 axis	^120^
Cancer	AT	circ-DB	Promoting HCC growth	Suppressing miR-34a and activating DB-related USP7	^38^
	ASCs	miR-122	Reducing HCC growth	Rendering HCC cells sensitive to chemotherapeutic agents and therefore increasing antitumor efficacy	^61^
	ASCs	miR-199a	Reducing HCC growth	Sensitizing HCC cells to doxorubicin by targeting mTOR and inhibiting mTOR pathway	^60^

AT, adipose tissue; BAT, brown adipose tissue; ATM, adipose tissue macrophage; ASC, adipose-derived stem cell; miRNA, microRNA; HDF, human dermal fibroblast; lncRNA, long noncoding RNA; MALAT1, metastasis-associated lung adenocarcinoma transcript 1; Klf4, Krüppel-like factor 4; MMP-9, matrix metalloproteinase-9; STAT3, signal transducer and activator of transcription 3; Bcl-2, B cell lymphoma-2; FGF21, fibroblast growth factor 21; DLL4, delta-like ligand 4; circ-DB, circular RNA related to deubiquitylation (has_circ_0025129); AMI, acute myocardial infarction; EGR1, early growth response factor 1; HCC, hepatocellular carcinoma; TRPM7, transient receptor potential melastatin-subfamily member 7; USP7, ubiquitin-specific protease 7; UTR, untranslated region; TLR4, Toll-like receptor 4; mTOR, mechanistic target of rapamycin

**Table 2:** Abbreviation table

Abbreviations	Full description
ASCs	Adipose-derived stem cells
ATMs	Adipose tissue macrophages
Ago2	Argonaute 2
AMI	Acute myocardial infarction
ADicerKO	Adipose-tissue-specific knockout of the miRNA-processing enzyme Dicer Arrestin domain-containing protein 1
ARRDC1	
ALIX	ALG2-interacting protein X
BAT	Brown adipose tissue
CEBPα	CCAAT/enhancer binding protein-alpha
circ-DB	Circ-deubiquitination
circRNAs	Circular RNAs
DLL4	Delta-like 4
EVs	Extracellular vesicles
Exo-ATMs	Exosomes derived from ATMs
EV-ASCs	EVs derived from adipose-derived stem cells
Exo-ASCs	Exosomes derived from ASCs
EGR1	Early growth response factor 1
ESCRT	Endosomal sorting complex responsible for transport
GDNF	Glial cell line-derived neurotrophic factor
HES1	Hairy and enhancer of split-1
HUVECs	Human umbilical vein endothelial cells
H/SD	Hypoxia/serum deprivation
HCC	Hepatocellular carcinoma
ILVs	Intraluminal vesicles
IFNγ	Interferon-γ
IBD	Inflammatory bowel disease
KLF7	Kruppel-like factor-7
Klf4	Krüppel-like factor 4
LncRNAs	Long noncoding RNAs
MSCs	Mesenchymal stem cells
MVs	Microvesicles
MVBs	Multivesicular bodies
mRNAs	Messenger RNAs
miRNAs	MicroRNAs
NSUN2	NOP2/Sun domain family member 2 protein
piRNAs	PIWI-interacting RNAs
PPARγ	Peroxisome proliferators-activated receptors-gamma
PUM2	Pumilio2
qPCR	Quantitative polymerase chain reaction
RISC	RNA-induced silencing complex
RBPs	RNA-binding proteins
snoRNAs	Small nucleolar RNAs
TSG101	Tumor susceptibility gene 101
TCF4	Transcription factor-4
TRADD	TNF receptor type 1-associated death domain protein
TNFα	Tumor necrosis factor alpha
TNFR1	Tumor necrosis factor receptor 1
TLR4	Toll-like receptor 4
TRPM7	Transient receptor potential melastatin 7
tRNAs	Transfer RNAs
UTRs	Untranslated regions
VPS4	Vacuolar protein sorting 4
YB-1	Y-box-binding protein 1

### Metabolic disease

Obesity is a concerning health issue that is generally characterized by excessive fat mass and quantity accumulation, which contributes to the occurrence of an array of complications, such as type 2 diabetes mellitus, hyperlipemia, and nonalcoholic fatty liver disease.^95^ Moreover, studies have already connected these obesity-associated metabolic disorders with the dysregulation of adipose-tissue-derived molecules, especially EVs.^96^^,^^97^ Importantly, the secretion of adipocyte-derived EVs was increasingly enhanced under circumstances of chronic, low-grade metabolic disorders associated with obesity, which was stimulated by inflammatory cytokines, such as TNF-α and interferon-γ (IFN-γ).^98^ Therefore, adipose-derived EVs can be regarded as messengers of metabolic responses related to the pathophysiological state of obesity. Furthermore, the RNAs in adipose-derived EVs are now considered novel adipokines engaged in such metabolic regulation.^34^ For instance, Thomou et al.^91^ first found exosomal miRNAs were significantly decreased in the serum of the ADicerKO mice, which endured severe metabolic disorders. Transplantation of BAT to the ADicerKO mice effectively restored the amount of circulating exosomal miRNAs and mitigated glucose intolerance with the level of fibroblast growth factor 21 (FGF21), a hormone that can regulate liver metabolism, remarkably reducing its circulation. Furthermore, miR-99b was highly expressed in BAT-derived exosomes and performed as a regulator in the expression of FGF21 in hepatocytes. Collectively, these findings implied the effects of brown adipocyte-derived exosomal miRNAs are adipokines that maintain metabolic homeostasis through transporting miR-99b and restraining production of FGF21.^91^

Furthermore, as macrophages are well known to influence inflammation and insulin sensitivity, emerging evidence has explored important exosome-mediated crosstalk between adipose-derived EVs and macrophages that regulate immune and metabolic homeostasis.^99^^,^^100^ Ying et al.^6^ proved that ATMs from obese animals produced miRNAs from exosomes that could result in insulin resistance and glucose intolerance both *in vitro* and *in vivo*. Furthermore, miR-155 was found to be differentially expressed in obese Exo-ATMs, which could suppress insulin signaling and glucose tolerance by directly inhibiting its target gene PPARγ.^6^ Pan et al.^101^ reported that adipocyte-secreted exosomes could transport miR-34a to ATMs. Then, expression of Krüppel-like factor 4 (Klf4) and the polarization of M2 were both repressed, thereby exacerbating metabolic dysregulation and obesity-induced inflammation. Of note, they found that, after a high-fat feeding, adiponectin, a traditional adipokine positively related to insulin-sensitizing activity, was more-obviously upregulated in miR-34a-knockout mice than it was in wild-type littermates. Moreover, the differential expression of adiponectin between these two groups disappeared after macrophage ablation, indicating that miR-34a had inhibiting effects on the production of adiponectin by indirectly modulating the quality of the macrophages.^101^^,^^102^ Collectively, these findings have offered new aspects of the complicated signaling mechanisms that connect RNAs of adipose-derived EVs and obesity-related metabolic diseases. Provided that these RNAs have been applied as specific biomarkers or tools, EV-based therapy may be much enhanced as a novel method of treating metabolic diseases.

### Cutaneous healing and regeneration

Optimal cutaneous wound healing requires a well-arranged integration of the complicated biological and molecular regulation of cell proliferation and migration and of extracellular matrix aggregation and remodeling.^103^ Adipose tissue as an endocrine organ secretes a multitude of kinds of adipokines to regulate distant targets, including skin.^104^^,^^105^ Previous studies have shown that both the ASCs and adipose tissue lipoaspirate extracellular fractions display therapeutic potential in wound healing.^74^^,^^106^ Moreover, as an indispensable component of extracellular fractions, adipose-derived EVs have also been demonstrated to take part in cutaneous wound healing.^107^^,^^108^ Studies have gone further to uncover the underlying biomolecular mechanisms of wound healing, which partly refer to the exact RNAs transferred by EVs. For example, Cooper et al.^74^ found that Exo-ASCs containing the lncRNA MALAT1 activated cell migration of human dermal fibroblasts *in vitro* and ischemic wound healing *in vivo*, whereas exosomes isolated from MALAT1-depleted conditioned medium were incapable of increasing cell migration. Another study^109^ illustrated that highly expressed miR-21 in Exo-ASCs could significantly accelerate the wound healing process and promote the proliferation and migration of HaCaT cells, which increased expression levels of matrix metalloproteinase-9 (MMP-9) in the PI3K/AKT pathway. In addition, Choi et al.^110^ performed an extensive analysis of miRNA expression between ASCs and Exo-ASCs and found Exo-ASCs contained many miRNAs (for example, miRNA-135a-5p, miRNA-378h, miRNA-586, and miRNA-1972) that could be related to fibroblast proliferation, ultraviolet protection, collagen synthesis, DNA repair, and cell aging, thus contributing to cutaneous restoration.^110^ To verify the microarray analysis, they showed that Exo-ASCs were able to alter important aspects of ultraviolet-B-damaged human dermal fibroblasts, including restoring their migration ability, reducing expression of MMPs, and increasing dermal matrix deposition *in vitro*.^111^ An aberrant overproduction of MMPs will accelerate skin photoaging because of their increased collagenolytic activity, which causes an imbalance between synthesis and degradation of the extracellular matrix, leading to excessive degradation of interstitial collagen.^112^ Therefore, considering the uncovered functions of miRNA contained in Exo-ASCs, the capacity of Exo-ASCs in controlling MMP activity is considered a new biochemical direction for skin recovery from photoaging. Apart from cell migration and proliferation, angiogenesis is also considered to have a critical role in wound healing and regeneration. Studies have indicated that Exo-ASCs can be absorbed by endothelial cells and then promote angiogenesis *in vitro* and *in vivo*.^113^ An additional study demonstrated that miR-125a was enriched in Exo-ASCs and could repress angiogenic inhibitor delta-like 4 (DLL4) to promote angiogenesis.^113^

Overall, studies of functional RNAs of adipose-derived EVs that provide meaningful insights into the intercellular communication and molecular mechanisms in the cutaneous regeneration process have mainly been conducted and verified in cell lines or primary cultures of keratinocytes, fibroblasts, or endothelial cells. There is no denying that these results may contribute greatly to new, promising, wound-healing therapies. Given the intricate cellular processes, diverse varieties, and multifactorial mediation of skin wounds, additional research related to cutaneous healing is needed to go deeply and profoundly into exploring different RNA functions in adipose-derived EVs.

### Parenchymal organ repair and regeneration

As mentioned, in a growing number of parenchymal organ diseases, associated RNAs in adipose-derived EVs have been identified as endocrine factors that participate in those pathophysiological processes. In addition, the crosstalk between adipose tissue and other tissues or organs via RNAs have provided novel potential therapeutic applications for treatment of related diseases.

In the cardiovascular system, many miRNAs, such as miR-22-3p, miR-29c-3p, miR-26a-5p, and miR-125b-5p, have been found in EV-ASCs and are associated with cardioprotective properties.^114^ AMI is considered as one of the most common causes of death throughout the world.^115^ Studies have indicated that adipose-derived EVs could act as target RNA-transfected vehicles to mediate AMI-related therapy.^62^^,^^116^ For instance, Liu et al.^117^ demonstrated that miR-93-5p-enhanced Exo-ASCs had better protective effects on infarction-induced myocardial damage than natural exosome treatment had, whereas *in vitro* results showed that miR-93-5p could exert an inhibiting effect on hypoxia-induced autophagy and inflammatory cytokine expression by acting on autophagy-related gene 7 (Atg7) and Toll-like receptor 4 (TLR4), respectively.^117^ Another similar study suggested that miR-146a-modified Exo-ASCs reduced AMI-induced myocardial damage by interacting with the 3′ UTR of early growth response factor 1 (EGR1) and suppressing expression of posttranscriptional EGR1 *in vivo* and *in vitro*.^62^ Research has also uncovered the important role of RNAs in the small EV (sEV)-mediated communication between adipose tissue and the liver.^118^ For example, lncRNA H19 released by EV-ASCs were proven to have a therapeutic role in acute liver failure by increasing the viability and proliferation of rat hepatocytes.^37^ Furthermore, in comparison with the treatment effects of EVs generated from non-silenced ASCs, the survival rate was significantly decreased when applying the coding sequence H19-silenced EV-ASCs to the treatment of rats with liver failure.^37^ Another study demonstrated that miR-181-5p-modified Exo-ASCs had a therapeutic effect on a liver fibrosis model. miR-181-5p-modified Exo-ASCs can downregulate Stat3 and Bcl-2 and stimulate autophagy in mouse hepatic stellate cells *in vitro* and reduce liver injury by downregulating collagen I, vimentin, a-SMA, and fibronectin in CCl4-induced liver fibrosis *in vivo*.^59^ In addition, because obesity can increase the risk of inflammatory bowel disease (IBD), Wei et al.^119^ found that the miRNA expression profile of visceral adipose exosomes was altered by a high-fat diet, which switched the exosomes from an anti-inflammatory phenotype to a pro-inflammatory phenotype. Furthermore, they revealed that circulating of exosomes from the obese fat transferred pro-inflammatory miR-155 to the colon, which promoted intestinal inflammation through accelerating macrophage M1 polarization. In addition, the exosome-mediated miR-155 inhibitor could prevent colitis as well.^119^ In the brain, the RNAs of adipose-derived EVs have been shown to enhance cerebral vascular remodeling after a stroke. The expression levels of miR-212-5p and miR-181b-5p were largely promoted in Exo-ASCs after ASCs were subjected to the brain extracts from rats with middle cerebral-artery occlusion, which showed that miR-212-5p and miR-181b-5p might be engaged in the development of an ischemic stroke. Additional studies have confirmed that, after oxygen-glucose deprivation treatment, the angiogenesis of brain microvascular endothelial cells was largely increased by Exo-ASCs through miR-181b-5p/transient receptor potential melastatin 7 (TRPM7) axis *in vitro*.^120^ Collectively, the RNAs of adipose-derived EVs have shown strong potential for clinical application in treating a few parenchymal organ diseases. Nevertheless, the organ disease models, which were proven to be effectively treated by EVs to date, have been restricted to organs or systems closely associated with adipose tissue, such as the liver and the cardiovascular system. Additional research related to other parenchymal organs is needed to fully disclose the RNA-mediated effects transferred by adipose-derived EVs.

### Cancer

There is also growing interest in remolding RNAs of adipose-derived EVs into cancer therapeutic drugs. It appears that the RNAs of adipose-derived EVs have conflictive functions in regulating tumor progression, recognized as both promoting and suppressing effects in different types of tumors. For example, studies have demonstrated that the migration and proliferation of human breast carcinoma cell line MCF7 was significantly promoted by Exo-ASCs by activating Wnt/β-catenin signaling.^121^ Moreover, as we mentioned, circ-DB secreted by Exo-AT was found to be enriched in patients with HCC and larger fat ratios. Further studies have demonstrated that exosomal circ-DB enhances HCC growth by suppressing miR-34a and activating deubiquitination-related USP7, which positively affects tumor growth and results in poor survival rates.^122^ In addition, the effects of Exo-AT on HCC cells could be reversed by knockdown of circ-DB.^38^ Collectively, those results provide certain bright prospects for understanding the promotional effects of adipose tissue on malignant tumors.

In contrast, adipose-derived EVs were demonstrated to perform as tumor inhibitors. Lou et al.^61^ showed that miR-122, which has been proven to link with HCC development and progression, was successfully transfected in Exo-ASCs.^61^, ^123^ The chemosensitivity to chemotherapeutic agents of cultured HCC cells was increased after uptake of these exosomes, and intra-tumor injection of these exosomes significantly increased the antitumor efficacy of chemotherapeutic agents *in vivo*, causing reduction in tumor growth.^61^ Similar research constructed miR-199a-modified Exo-ASCs, which could efficiently transfer miR-199a to HCC cells. Of note, miR-199a-modified Exo-ASCs remarkably sensitized HCC cells to doxorubicin by suppressing the mTOR pathway. Furthermore, intravenously injected miR-199a-modified Exo-ASCs dispersed to tumor tissues and significantly promoted the anticancer effects of doxorubicin against HCC *in vivo*.^60^ In summary, these studies undoubtedly indicate a novel anti-tumor clinical strategy of EV-ASCs via encapsulating either natural or engineered tumor-suppressing RNAs. However, new intense efforts are still needed to exploit the RNA-related functions and mechanisms of adipose-derived EVs in additional cancer types.

## Perspectives

Adipose-derived EVs have gradually drawn growing attention and have displayed several potential applications because of their extensive range of sources and easy availability and because they act as endocrine factors.^10^^,^^16^ As mentioned, adipose tissue and adipose-derived stem cells secrete abundant RNA molecules into the blood circulation via extracellular vesicle trafficking. These RNAs are thought to participate in various intercellular communications, which promote target-organ crosstalk between adipose tissue, such as skin, liver, colon, and the cardiovascular system. Of note, because advanced microarray analyses and RNA-sequencing can accurately uncover the specific type and relative intensity of RNAs that are selectively recruited into EVs, it becomes much easier to investigate functional verifications and conduct mechanism experiments of RNAs. Additionally, recent novel studies, which used engineered adipose-derived EVs loaded with target RNAs, could further expand the clinical application of RNA-based EV therapeutics.

Although prospects seem bright, several problems remain to be discovered and overcome. First, the existence of contaminating RNAs of multiple lipoproteins or RNA-binding proteins is rarely studied.^124^ Certain EV-isolation methods may be not be sufficient for wiping out the contamination of an EV-free RNA cargo, which will, of course, interfere with subsequent experiments of so-called EV-contained RNAs. For example, Karttunen et al.^125^ demonstrated that a precipitation-based EV-isolation method (e.g., the Invitrogen Total Exosome isolation) removed only portion of the plasma-vesicle-free miRNAs, which interferes with interpreting previous published data. Hence, further improvement of EV-isolation methods and identification of specific markers that show precisely the quality of the EV isolation and its purity are urgently required.

Another consideration comes from the RNA molecules themselves. RNAs in adipose-derived EVs contain a portion of non-coding RNAs, ranging in length from tens to thousands of nucleotides; however, target binding often involves partial complementarity with only a short length of nucleotides.^126^ When we artificially alter the amount of a certain novel RNAs in clinical trials, we should also pay attention to its targeting specificity, which may increase the possibility of unexpected and false conjunctions between RNAs and result in potential adverse effects in RNA-based EV therapeutics.^2^ Hence, detailed and careful assessments of any effects caused by target EV-contained RNAs are required in future clinical trials.

Last, but not least, adipose tissue is generally composed of multiple kinds of cells, such as mature adipocytes as well as a variety of stromal cells.^16^ The actual cellular sources of specific RNAs transferred by EVs as well as the relevant target pathways and tissues remain to be explored. In addition, many studies on the role of RNAs of adipose-derived EVs in different organ crosstalk have been rather preliminary. Further intensive mechanistic and biosafety studies are required to better and comprehensively understand the role of RNAs transferred by EVs to be able to develop the potential of their clinical applications.
